# Updates on therapeutic targeting of diguanylate cyclase for addressing bacterial infections: A comprehensive review

**DOI:** 10.1007/s11274-025-04512-6

**Published:** 2025-08-26

**Authors:** Roshan Mukund Tawale, Rafwana Ibrahim, Jesil Mathew Aranjani

**Affiliations:** Department of Pharmaceutical Biotechnology, Manipal College of Pharmaceutical Sciences, Manipal Academy of Higher Education, 576104 Manipal, India

**Keywords:** Diguanylate cyclase, C-di-GMP, Biofilm, Antimicrobial resistance, Antivirulence, Small-molecule inhibitors, Bacterial infections

## Abstract

The current global health issue of antimicrobial resistance necessitates innovative strategies for treating bacterial infections. A promising novel therapeutic target is the multisubunit diguanylate cyclase (DGC), which synthesizes cyclic di-GMP (c-di-GMP) and is implicated in biofilm formation. This multisubunit enzyme regulates critical virulence-associated behaviors in bacteria, such as biofilm formation, motility, and virulence factor synthesis, which are critical for biopathogenicity. This review focuses on the structural and functional characterization of DGCs, their contributions to bacterial pathogenesis, and recent advances in therapies targeting these enzymes. We describe innovations in small-molecule (SM) and peptide-based therapeutics and novel drug delivery platforms that alter DGC activity. In addition, we discuss new findings regarding DGCs and combination therapies of DGC inhibitors with other antibiotics. Finally, we outline the problems and prospects of therapies targeted to DGCs in the clinic. Inhibitors of DGCs may benefit from recent advances in structural biology techniques and medicinal chemistry approaches, which present new drug development opportunities.

## Introduction

Antimicrobial resistance (AMR) is a major global health crisis that has been listed by the World Health Organization (WHO) as one of the top 10 threats to global health (Jee et al. 2018). Traditional antibiotics exert strong selective pressure by targeting important bacterial processes, hastening the evolution of resistance mechanisms (Baym et al. 2016). This alarming trend has initiated scientific research into alternative therapeutic options that could effectively treat bacterial infections without restricting resistance development to a minimal extent. Anti-virulence strategies that interfere with bacterial virulence mechanisms instead of growth or survival are promising alternatives to traditional antibiotics.

Bacterial second messengers coordinate many cellular processes, including virulence and stress adaptability (Hengge et al. 2016). Owing to its pivotal role in regulating bacterial life, particularly the transition from planktonic to sessile lifestyles, cyclic dimeric guanosine monophosphate (c-di-GMP) has garnered considerable attention (Kalia et al. 2013). The intracellular levels of c-di-GMP are precisely balanced by two competing enzymatic activities: diguanylate cyclase (DGC), which produces c-di-GMP directly from two molecules of GTP, and phosphodiesterase (PDE) activity, which hydrolyzes c-di-GMP. The equilibrium of the two enzymes represents bacterial life, with a high level of c-di-GMP usually enhancing biofilm assembly and decreasing motility, whereas low levels are favorable for planktonic growth and increased motility (Hengge et al. 2016; Sadiq et al. 2017).

Biofilms, which are more complex than simple bacterial aggregates, are communities of microorganisms structured in a self-secreted extracellular polymeric substance (EPS) matrix. Biofilms represent a significant threat in medicine because of their heightened resistance to antibiotics and host defense (Valentini & Filloux 2016). According to conservative estimates, biofilms are responsible for more than 65% of infections and 80% of chronic infections. Compared with their planktonic state, the biofilm protective environment can increase antibiotic resistance from 10–1000-fold (Høiby et al. 2015; Uruén et al. 2020). Owing to the importance of c-di-GMP in the biofilm development process, modulating DGCs, that is, targeting biofilm-specific DGCs, is a logical step in fighting biofilm-related infections (Ha & O’Toole 2015).

The therapeutic approach utilizing DGCs as intervention sites stems from several salient characteristics. The first significant feature is the absence of DGCs in mammals, eliminating off-target effects and toxicity concerns for healthy cells. The second feature is the effect of DGC alteration on bacterial virulence while not killing bacteria, which may lower the likelihood of resistance developing due to decreased selective pressure. The third feature is the absence of a ruthlessness limit on bacterial species, which arguably broadens the scope of derogatory virulence drugs that can be manufactured because of their shared DGC catalytic domains (Heindl et al. 2014; Lai et al. 2025).

This review comprehensively analyzes DGCs as therapeutic targets for addressing bacterial infections. We explore the structural and functional characteristics of DGCs, their importance in bacterial pathogenesis, and current strategies for therapeutic intervention. Additionally, we discuss the challenges faced in developing DGC inhibitors and future directions. By synthesizing recent advancements in our understanding of DGCs and their therapeutic targeting, this review aims to highlight the potential of DGC inhibitors as a novel class of antivirulence agents that could complement our existing antimicrobial arsenal.

## Structure and function of diguanylate cyclases

### Molecular architecture of diguanylate cyclases

**Fig. 1:**
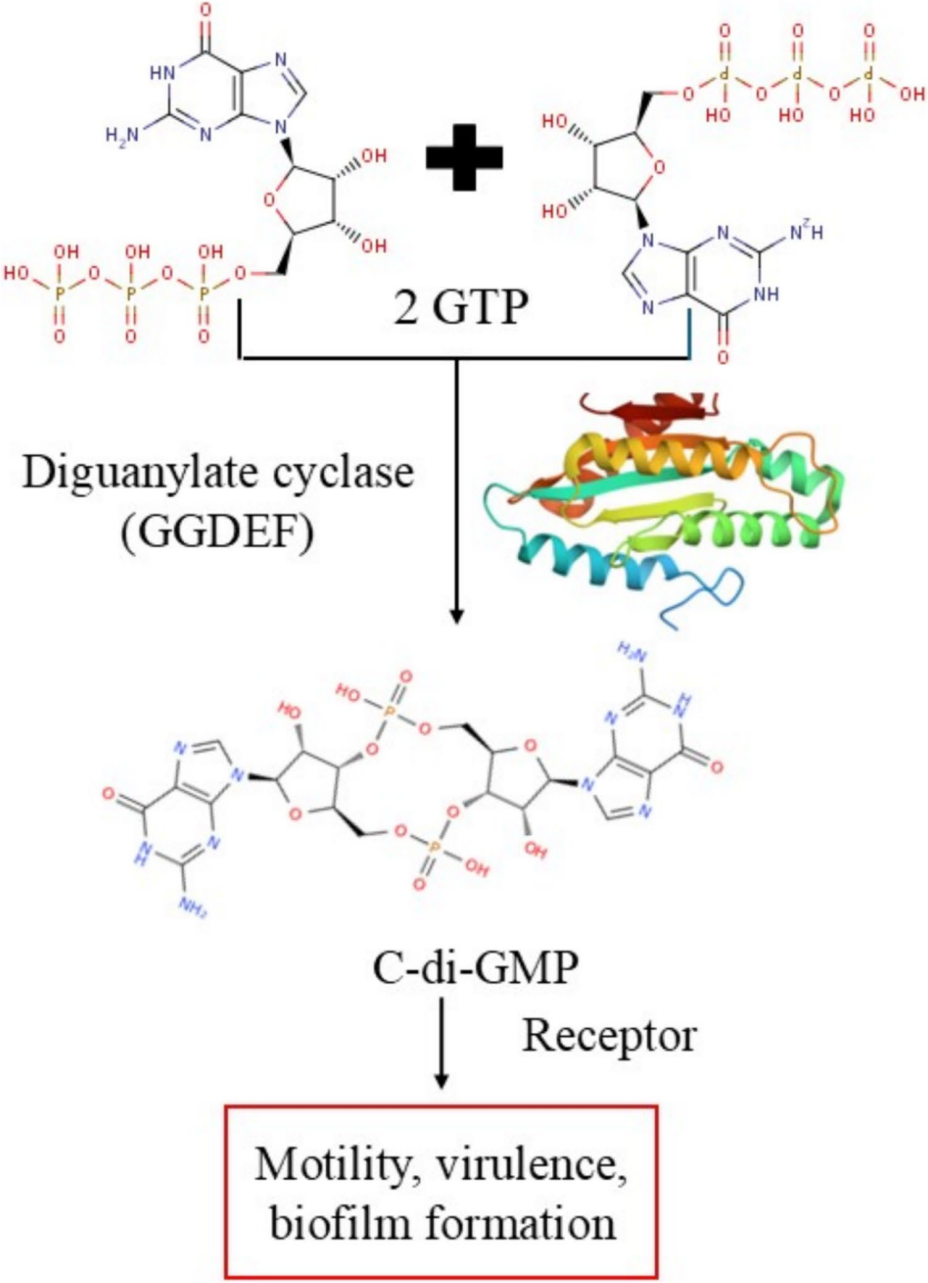
Schematic representation of the enzymatic synthesis of cyclic di-GMP (c-di-GMP) by a diguanylate cyclase (DGC) dimer. The ribbon diagram at the top illustrates the dimeric structure of the DGC enzyme. Two molecules of GTP bind to the active site in an antiparallel orientation. The catalytic mechanism involves a nucleophilic attack, which cyclizes the guanine nucleotides to form c-di-GMP and releases two molecules of pyrophosphate as byproducts. The chemical structures of GTP, c-di-GMP, and pyrophosphate are shown, highlighting the transformation during the reaction

Diguanylate cyclases are defined by the conserved GGDEF domain (named after the conserved amino acid sequence Gly-Gly-Asp-Glu-Phe), which contains a catalytic core responsible for c-di-GMP synthesis (Ryjenkov et al. 2005; Whiteley & Lee 2015). Structural studies have shown that functional DGCs exist as dimers and that each monomer contributes to the formation of an active site at the dimeric interface. The GTPs are docked to this binding region in an opposing parallel manner, which allows nucleophilic attack by the 3'-OH of one GTP to the α-phosphate of the other GTP, leading to c-di-GMP formation alongside the release of two pyrophosphate molecules (Whiteley & Lee 2015) (Fig. 1).

Many DGCs contain additional domains in addition to the catalyzing GGDEF, which is responsible for activity modulation and may respond to environmental changes. Some examples of these regulatory domains are PAS (Per-Arnt-Sim), GAF (cGMP-specific phosphodiesterases, adenylyl cyclases, and FhlA), HAMP (histidine kinase, adenylyl cyclase, methyl-accepting chemotaxis protein, and phosphatase), and REC (receiver) (Krasteva & Sondermann 2017; Whiteley & Lee 2015; Zschiedrich et al. 2016). Bacteria can modify their behavior in response to shifting conditions because of these domains, which allow DGCs to integrate various environmental signals and modify c-di-GMP synthesis appropriately.

Most DGCs have the distinctive feature of having an inhibitory or “I-site” apart from the active site. The I-site is usually equipped with an RxxD motif that binds c-di-GMP and exerts allosteric inhibition on the product (Christen et al. 2006; Hengge 2009). The additional synthesis of c-di-GMP is also inhibited after binding to the I-site because c-di-GMP constrains specific movements that limit the proper alignment of the GGDEF domains required for catalysis (Kuchma & O’Toole 2022; Schirmer & Jenal 2009). This feedback inhibition mechanism, which prevents the uncontrolled accumulation of c-di-GMP, allows for the tighter regulation of c-di-GMP levels within the cell. The segmental control of the cellular level of c-di-GMP becomes possible because of this feedback inhibition mechanism, which also prevents the excessive accumulation of c-di-GMP. Structural analysis and biomolecular techniques such as X-ray crystallography have provided important clues for understanding the catalytic mechanism of DGCs (Kalia et al. 2013; Schirmer 2016).

The crystal structures of DGCs such as *PleD* from *Caulobacter crescentus* and *WspR* from *Pseudomonas aeruginosa* show the distinct positioning of the catalytic residues and the conformational position of the constituents associated with activation and inhibition (Malone et al. 2007). These structural insights have been crucial in understanding the molecular frameworks that underlie DGC regulation and turning rational approaches into practical designs of DGC inhibitors.

### Regulation of diguanylate cyclase activity

Complex regulatory mechanisms at several levels govern DGC activity, guaranteeing accurate control of c-di-GMP synthesis in response to various environmental stimuli (Hengge 2009). Some of these regulatory mechanisms are allosteric regulation, spatial sequestration, and transcriptional and posttranslational control. Transcriptional regulation of DGCs controls gene expression in response to environmental stimuli. For example, in *P. aeruginosa,* the expression of several DGCs is controlled via quorum sensing along with the availability of nutrients during stress. This mechanism allows an organism to limit c-di-GMP and the cellular processes needed to adapt to the environment (Ha & O’Toole 2015; Valentini & Filloux 2016).

Other forms of modification at the molecular level, such as phosphorylation and proteolysis, bring another level of DGC modulation, which is represented mainly by posttranslational modifications. For example, cleavage of the N-terminus of the receiver domain allows the active form of PleD from *C. crescentus* to be transformed into dimers via its catalytic function along with dephosphorylation, which drives phosphorylation (Aldridge et al. 2003; Hecht & Newton 1995). DGCs can alter the levels of c-di-GMP through proteolytic decay, as observed with the regulation of *YcgR* in *E. coli*, whose abundance is regulated by ClpXP-mediated proteolysis (Hengge 2011; Pesavento 2011).

As described earlier, a feedback inhibition mechanism is achieved through allosteric regulation through the I-site (Christen et al. 2006). In addition, the binding of small-molecules or proteins to their regulatory domain regulates some DGCs. For example, the PAS domain in DosC from *E. coli* binds oxygen, linking oxygen availability to c-di-GMP synthesis and biofilm formation (Krasteva et al. 2012; Tuckerman et al. 2009; Wu et al. 2024).

Localization of DGCs to specific subcellular compartments involves the spatial regulation of DGCs, which influences their access to substrates, interaction partners, and downstream effectors. For example, in *P. aeruginosa*, *SadC* and DGC are localized to the inner membrane, where they regulate biofilm formation and virulence. The subcellular localization of DGCs can be either dynamic or responsive to environmental cues, which further adds to the complexity of c-di-GMP signaling networks (Guła et al. 2019; Zhu et al. 2016).

### Distribution and diversity of diguanylate cyclases in bacterial species

Genome analysis revealed extraordinary variation in DGCs across different bacterial species. The number of proteins containing the GGDEF domain is high in some species, which contain dozens of DGCs, whereas other species possess few or no DGCs (Randall et al. 2022; Römling 2023; Ryjenkov et al. 2005). A good example is *P. aeruginosa,* which contains approximately 40 GGDEF domain-containing proteins, indicating the abundance of c-di-GMP signaling networks in this adaptable pathogen (Feng et al. 2024; Merighi & Lory 2010). On the other hand, *Mycobacterium tuberculosis* contains only 1 DGC (Rv1354c), suggesting a simple c-di-GMP signaling system in the cell (Hong et al. 2013; Sharma et al. 2014).

The presence of multiple DGCs of a single species adds uncertainty to functional differentiation and the presence of redundancy. The evidence suggests that varying DGCs may control different cellular functions or work under particular environmental conditions. For example, in *P. aeruginosa*, *SadC* DGC is known to regulate swarming motility along with biofilm formation, and *WspR* controls the production of extracellular polysaccharides such as *Pel* and *Psl* (Merritt et al. 2007; Zhu et al. 2016). This efficient specialization is likely caused by differential expression, varying regulatory inputs, specific associations with downstream effectors, or specific interactions with controlling elements.

Phylogenetic studies have provided insights into the evolutionary history of DGCs, aiding in identifying conserved and divergent features. The GGDEF domain is conserved within DGCs, particularly the catalytic residues, whereas the regulatory domains are more diverse because of the need to process different environmental cues (Ryjenkov et al. 2005). This diversity in domain organization enhances the adaptability of c-di-GMP signaling pathways to regulate bacterial behavior across different ecological settings.

Recent studies have highlighted the importance of GGDEF domain proteins with degenerate active sites that lack enzymatic activity. These DGCs might bind GTP or c-di-GMP and act primarily as signal or scaffold receptors within the signaling cascade (Hengge 2009, 2021). The activity of these so-called ‘degenerate’ DGCs further complicates the understanding of c-di-GMP signaling pathways, as they may represent new pathways for therapeutic targeting.

### Crosstalk with other signaling pathways

C-di-GMP is not an independent mechanism but part of a network of pathways that integrate different bacterial signals to form a regulatory system that holistically manages different cell activities. The integration of c-di-GMP with other signaling systems, including quorum sensing, crosstalk, two-component systems, and other second messengers, allows bacteria to respond to various environmental stimuli.

Quorum sensing, a system of interactions and communication measured by cell density, involves c-di-GMP signaling and interfaces on many fronts. In the case of *P. aeruginosa*, quorum sensing modulates the expression of DGCs and PDEs controlled by LasR (Ledgham et al. 2003; Soto-Aceves et al. 2019). This phenomenon is associated with biofilm formation, population growth, and virulence factors. On the other hand, c-di-GMP can also control quorum sensing and set limits on producing or controlling the activity of quorum sensing regulators, thus creating a bidirectional regulatory interaction (Srivastava & Waters 2012; Valentini & Filloux 2019).

Another signaling layer that cooperates with c-di-GMP comprises sensor histidine kinases and a response regulator. Many response regulators with GGDEF domains can link the perception of the environment to the production of c-di-GMP. For example, the *GacS/GacA* two-component system in *P. aeruginosa* controls the expression of the small RNAs *RsmY* and *RsmZ*, which sequester the RNA-binding protein *RsmA*, which in turn controls the translation of several DGCs and PDEs (Brencic et al. 2009; Kay et al. 2006).

Various interactions occur between c-di-GMP signaling and other second messengers, such as cyclic adenosine monophosphate (cAMP), cyclic guanosine monophosphate (cGMP), and guanosine tetraphosphate (ppGpp). For example, in *E. coli*, ppGpp, the alarmone of the stringent response, inhibits the activity of certain DGCs, linking nutrient limitation to changes in c-di-GMP levels and biofilm formation (Hengge et al. 2016; Kalia et al. 2013).

Understanding thesecomplex signaling interactions is crucial for developing effective therapeutic strategies targeting DGCs. Disruption of specific nodes within these signaling networks may have broader effects on bacterial behavior than anticipated, which could be exploited for therapeutic purposes or lead to unintended consequences that must be carefully considered.

### Role of diguanylate cyclases in bacterial pathogenesis

#### Regulation of biofilm formation

Although several functions are associated with c-di-GMP, one of the well-established and researched fields is the regulation of biofilm formation, as biofilm formation is the main reason for bacterial persistence and antibiotic tolerance in numerous infections. Several underlying mechanisms through which the levels of c-di-GMP promote biofilm formation include the production of extracellular polysaccharides (EPSs), adhesins, and extracellular DNA, which collectively form the biofilm matrix.

C-di-GMP regulates the production of *Pel* and *Psl* polysaccharides in *P. aeruginosa*, a major cause of chronic lung infections in cystic fibrosis patients (Valentini & Filloux 2016). *Pel* and *Psl* are key biofilm matrix components that worsen disease conditions and therapy. Another DGC, *WspR*, responds mainly to surface contact and attachment, activating *Pel* polysaccharide production. Moreover, the DGC *SadC* influences both *Pel* and *Psl* synthesis (Merritt et al. 2007; Zhu et al. 2016). Mutants deficient in these DGCs exhibit impaired biofilm formation, highlighting their importance in this process.

Similarly, in *Escherichia coli*, c-di-GMP controls the production of cellulose and curli fimbriae, two major matrix components. The DGCs *YegE* and *YedQ* activate the cellulose synthase complex, whereas *YdaM* stimulates curli production through the transcriptional regulator CsgD (Brombacher et al. 2006; Da Re & Ghigo 2006). These examples illustrate how specific DGCs control distinct aspects of biofilm matrix production, allowing bacteria to fine-tune their biofilm architecture in response to environmental conditions.

In addition to affecting matrix production, c-di-GMP affects initial surface attachment, microcolony formation, and dispersal, among other aspects of biofilm development. The DGC The DGC CdgH is involved in the formation of microcolonies through as-yet-unidentified mechanisms. Moreover, DGC CdgD plays a role in facilitating initial attachment in *Vibrio cholera* by increasing the expression of the mannose-sensitive hemagglutinin (MSHA) pilus (Jones et al. 2015; Teschler et al. 2015; Zamorano-Sánchez et al. 2019). During the dispersal phase, a decrease in c-di-GMP levels, typically mediated by increased PDE activity rather than reduced DGC activity, triggers the release of bacteria from the biofilm, allowing the colonization of new sites.

#### Modulation of bacterial motility

C-di-GMP significantly controls bacterial motility and, in general, promotes a transition to a sessile lifestyle by particularly regulating multiple motility systems, including flagellar swimming, twitching mediated by type IV pili, and swarming. These regulatory effects occur mainly via transcriptional and post-translational mechanisms.

For example, in *Salmonella enterica* and *E. coli*, elevated c-di-GMP levels inhibit flagellar motility via the c-di-GMP-binding protein *YcgR*. By lowering swimming speed and motor function when it binds to c-di-GMP, *YcgR* interacts with the flagellar motor proteins MotA and FliG. Furthermore, the expression of flagellar genes can also be influenced by c-di-GMP, where high levels of c-di-GMP generally suppress the synthesis of flagellar components (Khan et al. 2020; Nieto et al. 2019; Zorraquino et al. 2013).

C-di-GMP also regulates type IV pili, which mediate twitching motility and aid in surface attachment. By influencing pilus extension and retraction, high c-di-GMP levels in *P. aeruginosa* prevent twitching motility (Valentini & Filloux 2016). The FimX protein, which binds c-di-GMP to modify pilus function, is involved in this regulation. It possesses degenerate GGDEF and EAL domains (Navarro et al. 2009).

High levels of c-di-GMP also inhibit swarming motility, a movement across surfaces that depends on flagella. Swarming in *P. aeruginosa* is suppressed when DGCs such as *SadC* and *RoeA* are overexpressed and enhanced when these genes are deleted (Merritt et al. 2007; Zhu et al. 2016). C-di-GMP inhibits swarming through direct interactions with flagellar function and indirect modulation of biosurfactant synthesis to promote surface motility.

The motility and c-di-GMP levels exhibit an inverse relationship during the microbial lifestyle transition from planktonic to sessile states. C-di-GMP ends the coordinated transition, allowing bacteria to better cope with changing environmental conditions by simultaneously repressing motility and inducing biofilm formation.

#### Impact on virulence factor production

Bacterial virulence is directly influenced by c-di-GMP due to its effects on biofilm development, motility, and virulence factors. Depending on the species of the organism, either high or low levels of c-di-GMP can lead to virulence.

When levels of c-di-GMP are high, virulence in *V. cholerae*, the causative agent of cholera, is reduced by limiting the expression of two important virulence factors: cholera toxin and TCP (toxin-coregulated pilus). The transcriptional regulator *VpsT*, which responds to c-di-GMP and downregulates the expression of *ToxT*, an essential virulence gene activator, is responsible for this regulation. Therefore, in animal models of cholera, increased virulence is generally associated with mutations that abate c-di-GMP levels (Hall & Lee 2018; Tischler & Camilli 2005).

Increased levels of c-di-GMP in *Bordetella pertussis*, the whooping cough agent, increase its virulence by promoting the expression of filamentous hemagglutinin and fimbriae, which adhere to respiratory epithelial cells (Dorji et al. 2018; Gutierrez et al. 2022). Like c-di-GMP, the expression of adhesins and other virulence factors associated with some *E. coli* strains that cause UTIs is positively controlled.

The impact of c-di-GMP on type III secretion systems (T3SSs), which deliver effector proteins directly into host cells, varies among bacterial species. In *P. aeruginosa*, high c-di-GMP levels typically suppress *T3SS* expression, which is consistent with the observation that biofilm-associated bacteria often exhibit reduced cytotoxicity compared with their planktonic counterparts. However, in *Salmonella enterica*, c-di-GMP can increase the expression of T3SS components under certain conditions, highlighting the context-dependent nature of c-di-GMP signaling (Pugazhendhi et al. 2022; Yuan et al. 2020).

#### Role in antimicrobial resistance and tolerance

C-di-GMP signaling helps bacteria resist and tolerate antimicrobial agents. This happens via multiple processes, including biofilm formation, efflux pump expression, and the stress response.

High levels of c-di-GMP help form biofilms that protect against antibiotics chemically and physically. The biofilm matrix restricts the diffusion of some antimicrobial agents. Moreover, biofilm-associated bacteria are in an altered physiological state. This means that the metabolism of these bacteria is reduced. Moreover, it also means increased stress responses. Thus, biofilm bacteria are more tolerant to antibiotics that target active processes (Lebeaux et al. 2014; Mah & O’Toole 2001; Roy et al. 2018; Uruén et al. 2020). Additionally, biofilms can facilitate horizontal gene transfer, accelerating the spread of resistance genes.

In addition to offering protection through biofilm formation, c-di-GMP also impacts the manifestation of resistance determinants in particular species. High levels of c-di-GMP increase the expression of the efflux pump MexAB-OprM in *P. aeruginosa*, which pumps out various antibiotics from cells. This contributes to inherent resistance. Similarly, c-di-GMP in *K. pneumoniae* regulates the expression of porins, which are membrane proteins that control the entry of antibiotics into the cell (Li et al. 2015; Yang et al. 2024).

C-di-GMP modulates bacterial stress responses to increase survival under antibiotic pressure. In *E. coli*, the c-di-GMP signaling pathway is involved in the general stress response pathway regulated by the alternative sigma factor RpoS, which regulates the expression of genes that protect against stress (Povolotsky & Hengge 2012). Furthermore, c-di-GMP influences the SOS response, a DNA damage repair system that enhances antibiotic tolerance and mutagenesis-driven resistance development.

C-di-GMP plays multiple roles in antibiotic resistance and tolerance. Therefore, targeting DGCs could be a fruitful therapeutic strategy. By reducing c-di-GMP levels, DGC inhibitors could increase the efficacy of conventional antibiotics by reducing biofilm formation, increasing bacterial susceptibility, and attenuating stress responses that contribute to tolerance.

#### Clinical significance in different bacterial infections

The clinical significance of c-di-GMP signaling extends across a wide range of bacterial infections, with particularly notable roles in chronic and biofilm-associated conditions. Understanding the specific involvement of DGCs in different infections is crucial for developing targeted therapeutic strategies (Table 1).

**Table 1:** Clinical significance of DGCs in various bacterial infections

Bacterial Species	Disease	Key DGCs	Clinical Significance	Reference
*Pseudomonas aeruginosa*	Cystic fibrosis lung infections	*WspR, SadC, RoeA*	Controls biofilm formation and antibiotic tolerance; mutations in DGCs are linked to mucoid phenotype	( Ha & O’Toole 2015; Malone et al. 2007; Merritt et al. 2007; Zhu et al. 2016)
*Vibrio cholerae*	Cholera	*CdgD, CdgH*	Regulates transition between planktonic (virulent) and biofilm states; affects colonization of intestinal mucosa	( Teschler et al. 2015; Zamorano-Sánchez et al. 2019)
*Escherichia coli*	Urinary tract infections	*YegE, YdaM*	Promotes biofilm formation on urinary catheters; enhances persistence in the urinary tract	( Da Re & Ghigo 2006)
*Mycobacterium tuberculosis*	Tuberculosis	Rv1354c	Contributes to dormancy and antibiotic tolerance; potential role in granuloma formation	( Hong et al. 2013; Sharma et al. 2014)
*Borrelia burgdorferi*	Lyme disease	*Rrp1*	Enhances survival during tick-to-mammal transmission; modulates host immune response	( Caimano et al. 2015)
*Klebsiella pneumoniae*	Pneumonia, UTIs	KPC_3301, KPC_2069	Promotes capsule production and hypervirulence; contributes to carbapenem resistance	( Karampatakis et al. 2023)

In lung infections, particularly those associated with cystic fibrosis (CF), phenotypic adaptation is observed in *P. aeruginosa*, which transitions from an acute virulent state to a chronic biofilm-forming state characterized by elevated c-di-GMP levels. The biofilm state of growth encases *P. aeruginosa*, shielding it from antibiotics and host immune defenses. This leads to enduring infections and progressive lung damage in CF patients. This adaptation is associated with mutations within DGCs and PDEs, with mucoid variants derived from CF patients frequently having modified c-di-GMP signaling. The biofilm mode of growth encases *P. aeruginosa* from antibiotics and the host immune defenses, contributing to the persistence of infections and progressive lung damage in CF patients (Sousa & Pereira 2014).

In *V. cholerae infections*, c-di-GMP signaling influences intestinal colonization and environmental persistence. While c-di-GMP enhances the expression of virulence factors, low levels during infection facilitate colonization of the small intestines (Conner et al. 2017). Nevertheless, once infection progresses, c-di-GMP promotes biofilm formation, as bacteria prepare for existence in ram ponds after host expulsion. This adaptable response from c-di-GMP signaling during the infection cycle emphasizes biphasic regulation.

In urinary tract infections (UTIs) caused by uropathogenic *E. coli* (UPEC), c-di-GMP regulates the formation of intracellular bacterial communities (IBCs), which are biofilm-like structures that protect bacteria from antibiotics and host defenses within bladder epithelial cells. Such IBCs add to the persistent and complex clinical issues associated with UTIs (Ravishankar 2024; Whelan et al. 2023). DGCs such as *YegE* and *YdaM*, which enhance intracellular bacterial communities, are other potential candidates that can help mitigate or control the problem of recurrent UTIs.

In the case of tuberculosis, the single DGC that *M. tuberculosis* encodes, RV1354c, appears to augment the pathogen’s capacity to enter into a dormant phase, where metabolic activity is substantially lowered, making it highly resistant to antibiotics. This state is said to describe latent tuberculosis infection, which is estimated to affect nearly a quarter of the globe’s population and can subsequently flare into an active disease. Concentrating on Rv1354c might address the challenges posed by latent tuberculosis, which currently does not have practical treatment approaches (Hong et al. 2013).

The clinical relevance of DGCs includes many other bacterial infections, such as those caused by *Borrelia burgdorferi* (Lyme disease) as well as those caused by *Klebsiella pneumoniae* (pneumonia and UTIs) and *Streptococcus pneumoniae* (pneumonia and otitis media). In all these situations, c-di-GMP signaling impacts critical stages in the pathogenesis of bacteria, including host colonization, immune evasion, and infection persistence, suggesting extensive treatment possibilities for targeting DGCs (Karampatakis et al. 2023).

### Therapeutic strategies targeting diguanylate cyclases

#### Small-molecule inhibitors

**Fig. 2:**
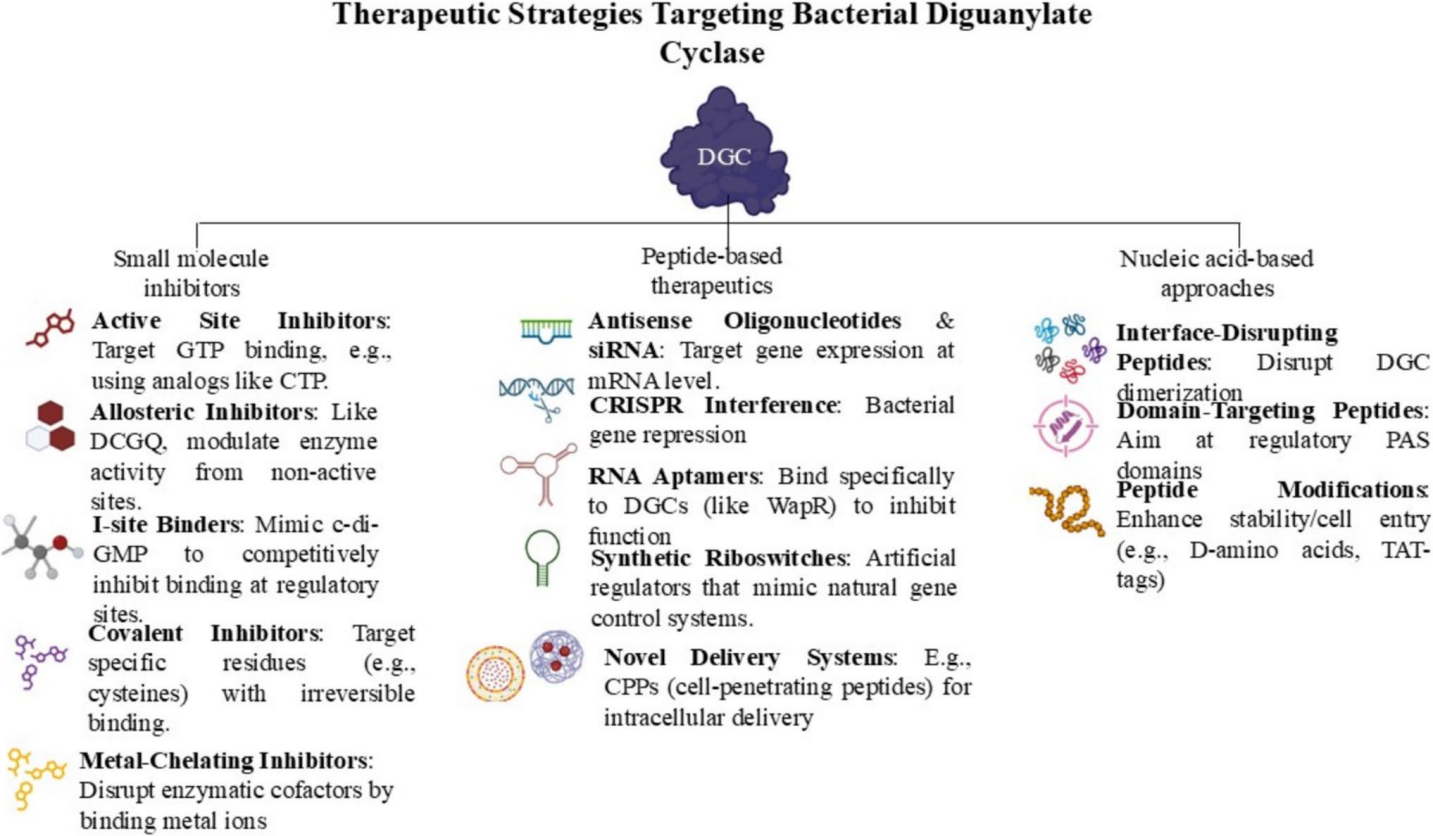
Therapeutic strategies targeting bacterial diguanylate cyclases (DGCs). This schematic categorizes current and emerging approaches to inhibit DGC activity and disrupt cyclic di-GMP (c-di-GMP) signaling pathways involved in bacterial biofilm formation and virulence. Strategies include small-molecule inhibitors (left), peptide-based therapeutics (center), and nucleic acid-based approaches (right), each targeting different aspects of DGC function, such as enzymatic activity, gene expression, dimerization, and domain-specific interactions

The most researched approach for DGCs is the use of small-molecule inhibitors because of their low cost, convenience of oral administration, and adaptability to enhance pharmacokinetic properties. As shown in Fig. 2, inhibitors can be categorized according to their binding locations and mechanisms.

Active site inhibitors block the incorporation of GTP by competing with it for the catalytic cavity of DGCs, thus directly inhibiting c-di-GMP synthesis. The active site of DGCs has several potential sites for the inhibition of GTP and non-nucleotide compound analogs. Promising sites are derived primarily from structure-based drug design (Manna et al. 2025; Vennard et al. 2025). Kyu Hon Cho et al. (2020) described a catechol-containing sulfonohydrazide with nanomolar affinity for the active site of *PleD* (Cho et al. 2020) (Table 2). Further studies revealed that these inhibitors bind directly to the catalytic site of *PleD*, not the I-site, and interact with the magnesium ion in the binding pocket. However, their effectiveness in reducing intracellular c-di-GMP levels in bacterial cells is limited, possibly due to membrane permeability issues. Similarly, thiazolidinedione derivatives act as *WspR*s from *P. aeruginosa* antagonists by binding to the GTP-binding pocket, decreasing biofilm formation in vitro and increasing antibiotic effectiveness in a mouse infection model (Froes et al. 2021; Lidor et al. 2015).

**Table 2:**
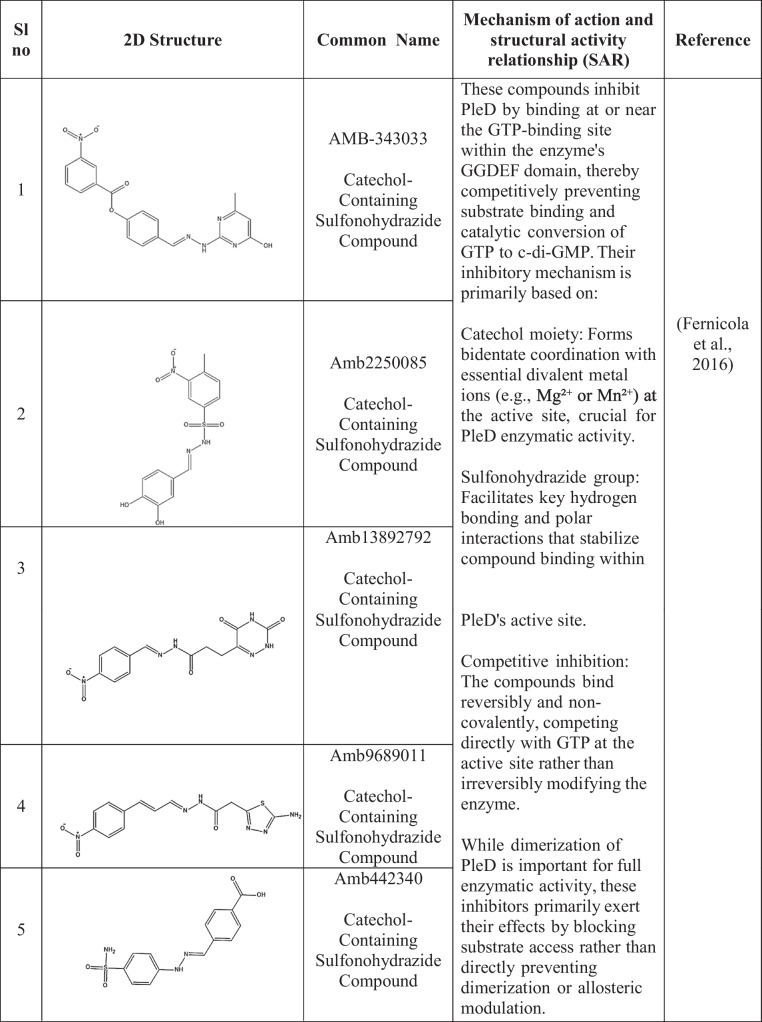

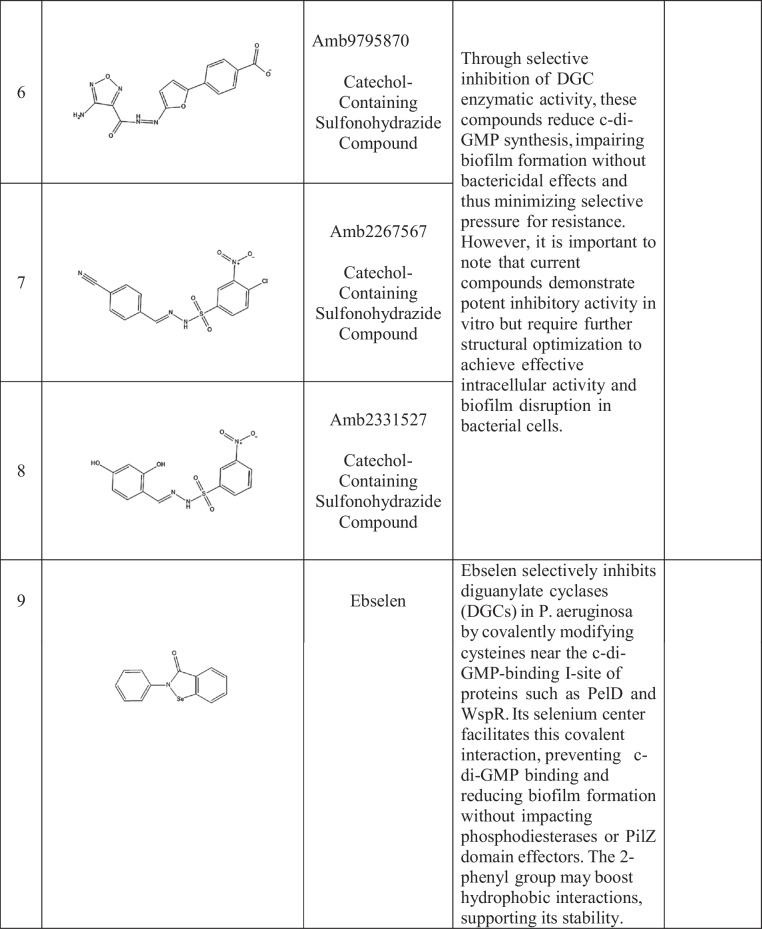

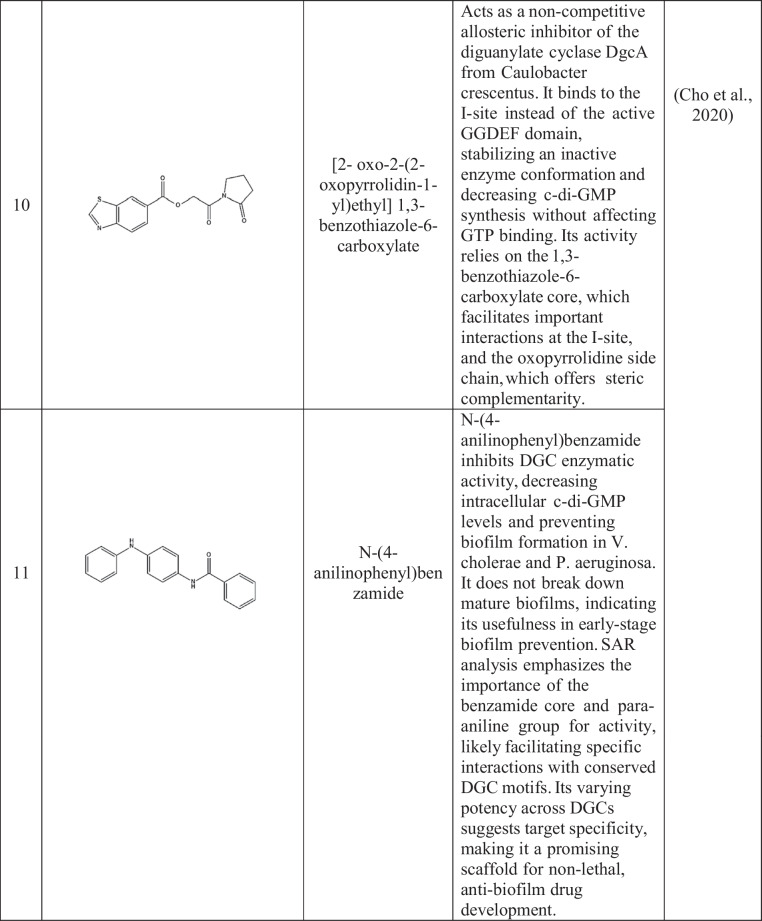

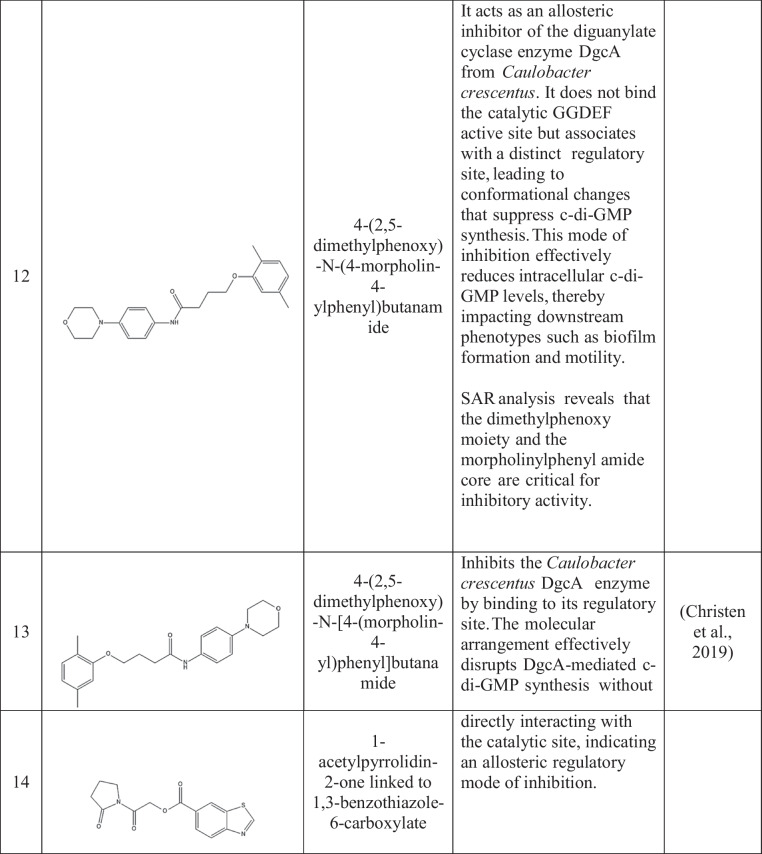
Small-molecule inhibitors of DGC

The enzymes show no activity when ligands inhibit them through allosteric feedback inhibition systems. Inhibitors attach to a location other than the active site, called the allosteric site, which hinders some conformations from suppressing catalysis. They prefer binding to an axis of two interconnected spheroid domains with different functions, such as a regulatory domain, thus enabling broader venue coverage in different DGCs.

I-site binders mimic the natural feedback inhibition mechanism by occupying the inhibitory site, which is typically an RxxD motif that interacts with c-di-GMP to regulate DGC activity (Christen et al. 2006; Dahlstrom et al. 2016; Kalia et al. 2013). Zhou et al. (2013) created synthetic c-di-GMP analogs with modifications at the 2'-OH position that demonstrated greater binding affinity to the I-site than did natural c-di-GMP, leading to increased inhibition of various DGCs. These analogs were shown to suppress biofilm formation in *P. aeruginosa*, indicating broad-spectrum activity (Zhou et al. 2013).

Covalent inhibitors bind covalently to some residues in diguanylate cyclases (DGCs), leading to irreversible inactivation. The approach can afford high potency and extended duration of action but requires optimization with care to avoid off-target effects. The compounds were effective against biofilm development by a diverse group of pathogens and were synergistic with conventional antibiotics (Lieberman et al. 2014; Wagner et al. 2016).

Metal-chelating inhibitors take advantage of the requirement of DGCs for divalent metal ions, e.g., Mg2 +, for catalysis. These inhibitors interfere with DGC activity through metal competition, chelation, or binding site occupation (Lieberman et al. 2014). Yang et al. (2023) reported that some hydroxamic acid derivatives chelate catalytic metal ions in DGCs, suppressing activity and biofilm formation in *P. aeruginosa* and *A. baumannii* (Olar et al. 2022; Wagner et al. 2016; Yang et al. 2023). Off-target interference with other metalloenzymes is a potential risk; however, careful optimization of these compounds is needed.

Recent developments in high-throughput screening technologies have significantly advanced the discovery of new DGC inhibitors. Christen et al. (2019) used a fluorescence-based screen to examine a library of more than 100,000 compounds. These authors identified several structurally diverse candidates capable of inhibiting various DGCs at submicromolar concentrations (Christen et al. 2019). Further research revealed that these compounds prevent biofilm formation and increase antibiotic sensitivity in *P. aeruginosa and E. coli*, indicating potential therapeutic benefits.

Despite these developments, some issues must be overcome before the clinical advancement of small-molecule DGC inhibitors. These issues include a need for selectivity against bacterial DGCs over human nucleotide-binding proteins, pharmacokinetic optimization of properties for sufficient tissue penetration, especially into biofilms, and prevention of the possible generation of resistance. Current research has attempted to overcome these issues by optimizing medicinal chemistry and investigating novel drug delivery routes.

#### Peptide-based therapeutics

Peptide-based therapeutics have several advantages for DGC targeting, such as high specificity, biodegradability, and the capacity to interfere with protein‒protein interactions, which could be challenging to target with small-molecules. Progress in peptide design and engineering has renewed interest in developing DGC inhibitors.

Interface-disrupting peptides are designed to target the dimerization interfaces of DGCs to prevent the formation of functional dimers necessary for catalytic activity (Dahlstrom et al. 2015, 2016; Spangler et al. 2011). Cyclic peptides can inhibit the dimerization of *PleD* by targeting its dimerization interface, which is activated by phosphorylation, as described in the study on *PleD* activation. This inhibition prevents cyclic di-GMP synthesis, disrupts biofilm formation and cellular differentiation, and offers a potential therapeutic approach to control infections regulated by the c-di-GMP signaling pathway (Paul et al. 2007).

Domain-targeting peptides can bind to specific regulatory domains of diguanylate cyclases (DGCs), influencing their responses to environmental stimuli. For example, a study on *Synechocystis sp*. PCC 6803 has shown that multiple PAS domains in *DgcA* are crucial for its full enzymatic activity, likely through their role in protein dimerization and stability. This highlights the potential of targeting PAS domains to modulate DGC function. Additionally, cyclic peptides, known for binding specific domains, offer a promising strategy for regulating DGCs such as *DgcZ* by selectively inhibiting or activating their activity in response to environmental changes (Ishikawa et al. 2020).

Peptide aptamers, short peptide motifs displayed on a scaffold protein, constitute another possible method for targeting DGCs (Li et al. 2019; Opoku-Temeng et al. 2016). The aptamers inhibited DGC activity in vitro and suppressed biofilm formation in *P. aeruginosa* and *S. aureus* when delivered through cell-penetrating peptide tags. As peptide aptamers can be easily adapted and optimized to target different DGCs, these peptides should be useful as tools to combat various bacterial pathogens (Elumalai et al. 2024; Ning et al. 2022)

Despite their potential, the application of peptide therapeutics is hampered by several drawbacks, such as low stability in biological fluids, membrane impermeability, and potential immunogenicity. Peptide modifications have been highly studied to overcome these drawbacks. Cyclization (e.g., via disulfide bridges or other types of bridging groups) stabilizes peptides and increases binding affinity by removing conformational flexibility (Gongora-Benitez et al. 2014). Stapled peptides, which contain a synthetic bridge connecting two amino acid side chains, adopt a stable helical conformation, making them more potent at disrupting protein‒protein interactions (Tsuchiya et al. 2022).

N-terminal acetylation, C-terminal amidation, and D-amino acid or noncanonical amino acid incorporation can significantly increase peptide stability against proteolytic degradation (Gentilucci et al. 2010). Jacobsen (2013) genetically encoded a peptide containing D-amino acids to bind to the regulatory domain of *SadC in P. aeruginosa* and showed increased stability in serum and greater efficacy in preventing biofilm formation in a murine model of lung infection than its L-amino acid counterpart (Jacobsen 2013).

Cell-Penetrating Peptides (CPPs) do exemplify the ability of peptide-based inhibitors and other biomolecules to penetrate bacterial membranes and achieve intracellular delivery (Hadjicharalambous et al. 2022). Widely used CPPs, such as TAT (from a transactivator of transcription from HIV), arginine-rich motifs, and amphipathic peptides, have been conjugated with DGC-targeting peptides to improve their cellular uptake (Guidotti et al. 2017; Huang & Li 2023). Recent advances in peptide display technologies, including phage display, mRNA display, and yeast surface display, have increased the identification of DGC-targeting peptides with improved binding affinity and specificity (Chen et al. 2013; Keefe & Szostak 2001). These technologies facilitate the screening of extensive peptide libraries (109–1013 individual peptides) for lead drug candidates with optimal characteristics. Together with rational design and computer modeling, these technologies have led to the development of peptide inhibitors with nanomolar binding affinities to some DGCs (Banta et al. 2013; Rothe et al. 2006).

#### Nucleic acid-based approaches

Nucleic acid-based strategies, including antisense oligonucleotides (ASOs), small interfering RNAs (siRNAs), and CRISPR interference (CRISPRi), offer innovative approaches to modulate the expression of diguanylate cyclases (DGCs) in bacterial pathogens. These methods aim to downregulate DGCs at the genetic level, thereby reducing the intracellular levels of cyclic di-GMP (c-di-GMP). This secondary messenger regulates biofilm formation, motility, and virulence in bacteria.

A notable study by Moustafa et al. (2021) demonstrated the efficacy of peptide-conjugated phosphorodiamidate morpholino oligomers (PPMOs) in targeting essential genes in *Pseudomonas aeruginosa* (Moustafa et al. 2021). These PPMOs, which function through antisense mechanisms, inhibited bacterial growth and biofilm formation both in vitro and in vivo. This study revealed that PPMOs targeting genes such as *acpP, lpxC,* and *rpsJ* were effective against multidrug-resistant strains of *P. aeruginosa*, suggesting their potential as therapeutic agents against biofilm-associated infections.

In addition to PPMOs, CRISPRi has been employed to regulate DGCs in bacterial pathogens. For example, a study by Noirot-Gros et al. (2019) utilized CRISPR interference to simultaneously repress multiple DGC genes in *P. aeruginosa*, significantly reducing biofilm formation. This approach capitalizes on the redundancy of DGCs in bacterial signaling pathways, providing a robust strategy to disrupt biofilm formation (Howard et al. 2017; Noirot-Gros et al. 2019).

While these nucleic acid-based strategies show promise, challenges remain concerning the delivery of these molecules into bacterial cells. Advancements in delivery systems, such as the development of lipid nanoparticles and the use of cell-penetrating peptides, are crucial to enhance the efficacy of these therapies. Moreover, the specificity of these approaches allows for targeted inhibition of individual DGCs, which can be advantageous in fine-tuning bacterial signaling pathways. However, this specificity may also limit the broad applicability of these strategies, necessitating the development of combination therapies that target multiple DGCs or integrate nucleic acid-based approaches with traditional antibiotics.

#### Drug delivery strategies for diguanylate cyclase inhibitors

Achieving efficient intracellular targeting of DGC inhibitors, especially within biofilms, represents an important obstacle for implementing clinically applicable therapies. Novel drug delivery approaches are under investigation to increase the bioavailability, stability, and targeting of DGC inhibitors and reduce the side effects of unwanted targets.

Crosslinking between protamine and DGC inhibitors decreases the ability of DGC inhibitors to aggregate in saltwater. The nanoparticle formulations of DGC inhibitors have several advantages, such as protection against degradation, increased cellular uptake, and sustained release profiles. Polymeric nanoparticles, consisting of biodegradable polymers, including poly (lactic‒co‒glycolic acid) or chitosan, have been employed to encapsulate diverse DGC inhibitors. Cho et al (2020) studied several potent DGC inhibitors, such as DC1058 and other small synthetic molecules, which exhibit low membrane permeability, limiting their antibiofilm activity when used in free form. Encapsulation into nanoparticles can improve their delivery across bacterial membranes and biofilm matrices, increasing their intracellular access and inhibitory effects on c-di-GMP synthesis (Birk et al. 2021; Cho et al. 2020; Makadia & Siegel 2011; Shariati et al. 2022). In addition to polymeric nanoparticles, nanodisc self-assembled phospholipid bilayers stabilized by scaffold proteins have been used to reconstitute membrane-bound DGC enzymes. While this is more relevant for biochemical assays than for drug delivery, it highlights the utility of nanoscale lipid assemblies in DGC-related research (Richter et al. 2020).

Liposomes are encapsulating structures formed by layers of fats that show great potential in delivering DGC inhibitors (Sharma et al. 2021). Liposomal delivery protects DGC inhibitors from degradation, improves their penetration into the biofilm matrix, and facilitates fusion with bacterial membranes, often aided by membrane-destabilizing peptides, enabling direct intracellular delivery of the inhibitors. This targeted approach effectively reduces intracellular c-di-GMP levels, disrupting biofilm formation and promoting biofilm dispersal more efficiently than free drugs do. Such liposomal systems provide sustained and controlled release of DGC inhibitors at infection sites, overcoming challenges such as poor membrane permeability and biofilm barriers, and have demonstrated superior biofilm eradication in experimental models of infections caused by pathogens such as *Pseudomonas aeruginosa* (Makhlouf et al. 2023; Panthi et al. 2024; Sharma et al. 2021).

Bacteriophage-based delivery systems use the phage’s ability to target bacteria and deliver genes specifically. Phage bioconjugates represent a promising strategy for enhancing the delivery and efficacy of diguanylate cyclase (DGC) inhibitors in bacterial pathogens, including *Pseudomonas aeruginosa*. These bioconjugates leverage the specificity and efficiency of bacteriophages to target bacterial cells, potentially overcoming challenges associated with traditional antibiotic therapies. A notable example is the development of fusogenic liposomes engineered to codeliver DGC inhibitors and membrane-destabilizing peptides. Compared with control liposomes and free drugs, these liposomes demonstrated superior performance, effectively fusing with bacterial membranes and facilitating the entry of the inhibitor into bacterial cells. This approach resulted in a more significant reduction in cyclic di-GMP levels, indicating enhanced efficacy in disrupting biofilm formation.

Additionally, bacteriophage-encoded peptides that interfere with the c-di-GMP signaling pathway in *P. aeruginosa* have been identified. These peptides, derived from PB1-like *Pseudomonas* phages, interact directly with the DGC *YfiN*, increasing the intracellular levels of c-di-GMP. This interaction affects various processes, including cellular motility and biofilm formation, suggesting a novel mechanism to manipulate bacterial signaling pathways (De Smet et al. 2021).

Furthermore, phage-derived depolymerases have been explored as adjuncts to antibiotic therapy. These enzymes degrade extracellular polysaccharides, such as γ-polyglutamic acid, which compose the protective biofilm matrix. By breaking down this matrix, phage-derived depolymerases enhance the penetration and efficacy of antibiotics, including DGC inhibitors, against biofilm-associated bacteria (Islam et al. 2024).

Bacterial outer membrane vesicles, or OMVs, are small particles that Gram-negative bacteria release naturally. These OMVs have a new role as carriers for antimicrobial agents, which are substances that kill or slow harmful bacteria. A study by Ramirez et al. (2023) demonstrated that OMVs derived from *Escherichia coli* Nissle 1917 could inhibit *P. aeruginosa* biofilm formation by downregulating key genes such as *algD* and *PpyR*, significantly reducing biofilm biomass. Compared with free drugs, the encapsulation of DGC inhibitors within OMVs enhances their stability, targeted delivery, and efficacy, enabling deeper penetration into biofilms and disrupting bacterial signaling pathways more effectively (Alaei et al. 2023). This technique proved to be better at breaking up biofilms, which are protective layers that bacteria create. The new method disrupted these biofilms more effectively than the free inhibitor did, even in different bacterial groups.

Stimuli-responsive delivery systems are engineered to release diguanylate cyclase (DGC) inhibitors specifically at infection sites, thereby enhancing therapeutic efficacy while minimizing systemic side effects. These systems exploit distinct microenvironmental cues prevalent in bacterial infections, such as acidic pH, the presence of bacterial enzymes such as lipases and proteases, and differential redox potentials between infected and healthy tissues.

For example, in 2023, researchers developed pH-sensitive nanoparticles that release DGC inhibitors under the acidic conditions characteristic of *Pseudomonas aeruginosa* biofilms. This targeted release mechanism improves the effectiveness of treatment against biofilms and reduces potential side effects in surrounding healthy tissues. Additionally, enzyme-responsive systems have been designed to release antimicrobial agents upon encountering specific bacterial enzymes, such as lipases, which are overexpressed during infection. These systems increase the precision of drug delivery and minimize off-target effects (Zhou et al. 2022).

Furthermore, nanoparticles responsive to reactive oxygen species (ROS) have been explored, leveraging the elevated ROS levels in inflamed tissues to trigger drug release. This approach ensures that therapeutic agents are activated precisely at the site of infection, thereby increasing their efficacy and reducing systemic exposure. These stimuli-responsive delivery systems collectively represent a promising strategy for treating bacterial infections, offering greater specificity and fewer side effects than conventional therapies do (Guo et al. 2023).

As stated previously, the delivery of autoimmune agents depends strongly on the characteristics of the targeted area. The chosen method of medicine delivery will depend on the characteristics of the inhibitor, the bacteria to be infected, the infection site, and the expected behavior of the medicine within the body. Combining these innovative delivery methods with scientifically developed DGC inhibitors offers hope for creating strong treatments for challenging biofilm infections that can be used in medical practice.

#### Combination therapies involving diguanylate cyclase inhibitors

Combining DGC inhibitors with standard antibiotics offers novel strategies for treating and managing biofilm-related infections. These combinations may surpass the unipolar approach by impeding both biofilm development and bacterial death, which optimally would result in greater effectiveness (Andersen et al. 2021).

Numerous authors have described the collaborative action of different antibiotics and DGC inhibitors. Wang et al. (2024) reported that QSIs can disrupt the bacterial communication systems responsible for biofilm formation and virulence factor production. While QSIs alone have limited antibacterial effects, their combination with antibiotics often results in synergistic activity that enhances bactericidal efficiency and reduces the development of resistance (Wang et al. 2024). Another study by Furiga et al. (2016) investigated the effects of a novel quorum-sensing inhibitor (QSI) called C11, which is structurally based on C4-homoserine lactone, on *P. aeruginosa* biofilms formed under both aerobic and anaerobic conditions. They reported that C11 significantly inhibited biofilm formation and downregulated key quorum-sensing regulatory genes (*rhl* and *las* systems) and virulence genes, reducing pathogenicity. Importantly, when combined with antibiotics such as ciprofloxacin, tobramycin, and colistin, C11 exhibited synergistic antibiofilm activity, increasing antibiotic efficacy and increasing biofilm susceptibility under both growth conditions, suggesting its potential to improve treatment outcomes in chronic infections such as cystic fibrosis-related lung disease (Furiga et al. 2016).

This multilayered reasoning of the synergism described above draws on the proofs cited above. These compounds reduce c-di-GMP levels by inhibiting DGCs, which leads to lower EPS production. This decrease in EPSs typically acts to limit antibiotic access into biofilms. Moreover, decreased c-di-GMP levels can affect bacterial physiological states and increase metabolism, increasing bacterial vulnerability to certain antibiotics that act on metabolically active cells. DGC inhibitors may also reduce some stress responses and tolerance mechanisms associated with biofilm antibiotic persistence.

The issues with clinical translation include possible pharmacokinetic interactions between combined agents and toxicity concerns from multiple compounds, even though they present promising results from preclinical studies. Optimized dosing schedules are also a challenge. Such interactions require complex studies on PK/PD and intricate designs of clinical trials to meet the defined objectives.

The development of more effective therapies could stem from the rational design of combination strategies based on the mechanistic comprehension of the c-di-GMP signaling system and antibiotic action to better treat biofilm-associated infections. For example, a combination of DGC inhibition, which serves as a target for biofilm formation, could resolve several hurdles to efficient antibiotic therapy.

#### Emerging technologies in diguanylate cyclase research and drug development

Recent technological advances have accelerated the understanding of DGCs and the development of therapeutics targeting these enzymes. These emerging technologies span various fields, including structural biology, computational methods, high-throughput screening, and innovative preclinical models (Table 3).

**Table 3:** Emerging technologies in DGC research and drug development

Technology	Application	Recent Advancements	Impact on DGC Research	Reference
Cryo-electron microscopy (Cryo-EM)	High-resolution structural analysis	Sub2 Å resolution of GGDEF domains; visualization of conformational changes	Detailed understanding of catalytic mechanisms and allosteric regulation	( Tang et al. 2023)
Single-molecule techniques	Real-time analysis of enzyme dynamics	FRET-based monitoring of DGC conformational changes; optical tweezers for force measurements	Insights into the kinetics and dynamics of DGC activation and inhibition	( Christen et al. 2019)
Fragment-based drug discovery	Identification of novel inhibitor scaffolds	MS-based screening; NMR-based fragment screening; X-ray crystallography	Discovery of novel binding sites and inhibitor chemotypes	( Martino et al. 2025)
Artificial intelligence (AI) and machine learning	Virtual screening and drug design	Deep learning models for predicting DGC inhibitors; AI-based analysis of structure–activity relationships	Accelerated identification of promising DGC inhibitors	( Fernicola et al. 2016)
Microfluidic systems	High-throughput screening and biofilm models	Droplet-based assays for DGC activity; microfluidic biofilm growth systems	Rapid screening of inhibitors under physiologically relevant conditions	( Katharios-Lanwermeyer et al. 2022; Lacanna et al. 2016)
CRISPR/Cas technologies	Genetic manipulation of DGCs	Base editing of DGC genes; CRISPRi for simultaneous silencing of multiple DGCs	Detailed understanding of DGC functions in bacterial pathogenesis	( Noirot-Gros et al. 2019)
Click chemistry	Covalent inhibitor development; Activity-based protein profiling	Bio-orthogonal reactions for DGC labeling; in situ chemistry for inhibitor optimization	Enhanced understanding of DGC activity in complex environments	( Fernicola et al. 2015)

Recent advances in cryo-electron microscopy (cryo-EM) have significantly transformed structural biology by enabling high-resolution visualization of proteins without crystallization. This includes detailed structural elucidation of the GGDEF domains and full-length diguanylate cyclases (DGCs). Single-particle cryo-EM techniques facilitate the capture of multiple conformational states of biomolecules, thereby providing critical insights into catalytic mechanisms and allosteric regulation, as highlighted by Zhong et al. (2023) (Tang et al. 2023). In addition to these structural approaches, single-molecule techniques such as Förster resonance energy transfer (FRET) have been instrumental in characterizing the conformational dynamics of DGCs, revealing complex, multistep activation pathways that inform the design of allosteric inhibitors.

Single-molecule techniques such as Förster resonance energy transfer (FRET) and optical tweezers have provided critical insights into the real-time dynamics of diguanylate cyclases (DGCs), including conformational changes during activation and inhibition. For example, FRET-based biosensors have been used to monitor c-di-GMP binding and DGC activity, as demonstrated in studies identifying small-molecule modulators of *Caulobacter crescentus* DgcA. These approaches revealed how slight chemical substitutions in inhibitors can switch allosteric regulation from inhibition to activation, highlighting the dynamic interplay between enzyme conformations and ligand interactions (Christen et al. 2019).

Another study by Fernicola et al. (2016) used a structure-based virtual screening approach to identify small-molecule inhibitors targeting the active site of the diguanylate cyclase *PleD* from *Caulobacter crescentus*. By employing a three-dimensional pharmacophore model and docking compounds from the ZINC database, they selected seven candidate molecules, two of which, catechol-containing sulfonohydrazide compounds, were found to competitively inhibit *PleD* at low micromolar concentrations (IC_50_ ≈ 11 μM) in vitro. These results prove that virtual screening can yield potent DGC inhibitors and support further development of such molecules as antibiofilm agents (Fernicola et al. 2016).

Fragment-based drug discovery (FBDD) has been successfully employed to identify nonnucleotide allosteric inhibitors of *PleD*, broadening the range of pharmacological strategies beyond traditional nucleoside analogs. Microfluidic platforms have enabled high-throughput screening of DGC inhibitors under physiologically relevant conditions, increasing the predictive power for in vivo efficacy. Furthermore, genetic tools such as CRISPR interference (CRISPRi) have been utilized to simultaneously repress multiple DGC genes in *Pseudomonas aeruginosa*, revealing the extent of functional redundancy and compensatory networks that complicate single-target inhibition approaches (Martino et al. 2025).

Advanced biofilm models, including 3D-printed microfluidic devices replicating the architecture and microenvironment of cystic fibrosis airways, have facilitated preclinical evaluation of DGC-targeted therapies under infection-relevant conditions. Additionally, biocompatible click chemistry has enabled the synthesis of covalent DGC inhibitors and activity-based probes, allowing real-time monitoring of enzymatic activity and target engagement within complex biological systems. Microfluidic systems have become essential tools for high-throughput screening of diguanylate cyclase (DGC) inhibitors and modeling biofilm growth under physiologically relevant conditions. Droplet-based assays and microfluidic biofilm growth platforms enable rapid, quantitative analysis of DGC activity and biofilm formation, efficiently identifying inhibitory compounds. For example, microfluidic devices have been used to monitor the effects of DGC mutations and inhibitors on *Pseudomonas aeruginosa* biofilm maintenance and cell viability, providing dynamic and reproducible environments for testing (Katharios-Lanwermeyer et al. 2022; Lacanna et al. 2016). These approaches facilitate the discovery and characterization of DGC inhibitors by mimicking infection-relevant conditions and enabling real-time observation of bacterial responses.

Applying these innovative technologies to DGC research and the drug development process holds great potential to accelerate the translation of findings from basic science to therapeutically beneficial results. Addressing primary obstacles in treating biofilm-associated infections, these technologies provide advanced knowledge on DGC structure and function, more efficient strategies for designing inhibitors, and better models for testing predictive efficacy.

### Insights from clinical and preclinical studies

#### Preclinical evaluation of diguanylate cyclase inhibitors

The preclinical evaluation of DGC inhibitors is a comprehensive and multistage process aimed at assessing the efficacy, safety, and translational potential of these compounds as antibiofilm and antivirulence agents. Diguanylate cyclases (DGCs) synthesize cyclic di-GMP (c-di-GMP). This ubiquitous bacterial second messenger regulates biofilm formation, motility, and virulence factor production. By modulating c-di-GMP levels, DGC inhibitors present a strategic approach to disarm pathogens without inducing bactericidal or bacteriostatic effects, potentially mitigating the selective pressure that drives antibiotic resistance (Allen et al. 2014).

Initial screening of DGC inhibitors typically employs traditional in vitro assays, such as microtiter plate-based crystal violet staining, to quantify biofilm biomass under static conditions. While applicable for preliminary assessments, these assays do not accurately reflect the complex physiological environments encountered in vivo, where factors such as fluid dynamics, nutrient gradients, and host immune responses can significantly impact biofilm formation and inhibitor efficacy. To address these limitations, more advanced models, including microfluidic flow cells and organ-on-chip systems, have been developed to better simulate the conditions of human infection sites. For example, airway-on-a-chip models that incorporate human bronchial epithelial cells cocultured with *Pseudomonas aeruginosa* biofilms can recapitulate the pathological milieu of cystic fibrosis lungs, providing a more clinically relevant platform for evaluating the impact of DGC inhibitors (Benam et al. 2016; Huh et al. 2010).

A significant concern with any antimicrobial strategy is the potential for resistance development. Unlike conventional antibiotics, which exert selective pressure by targeting essential bacterial processes, DGC inhibitors target virulence pathways without directly affecting bacterial viability. This antivirulence approach is postulated to impose less selective pressure for resistance emergence.

The evaluation of DGC inhibitors has progressed from in vitro biofilm assays to more complex ex vivo and in vivo models. In vitro models, ranging from static systems to dynamic flow cells, enable the assessment of biofilm inhibition and dispersal under controlled conditions. Sambanthamoorthy et al. (2014) reported that small-molecule DGC inhibitors effectively reduce biofilm formation by pathogens such as *P. aeruginosa* and *Acinetobacter baumannii* in static and flow cell systems. Additionally, these inhibitors have demonstrated efficacy in dispersing preformed biofilms on clinically relevant surfaces, including urinary catheters (Sambanthamoorthy et al. 2012, 2014).

Ex vivo models, which utilize human airway tissues or patient-derived samples, offer further insights into the clinical potential of DGC inhibitors. While direct studies using patient-derived organoids are limited, the application of organ-on-chip platforms and ex vivo tissue models is gaining traction for personalized infection research and targeted drug screening (Benam et al. 2016).

Animal models remain indispensable for preclinical evaluation, providing a means to assess the in vivo efficacy of DGC inhibitors in the context of infection. Mouse and rat models of respiratory, urinary tract, and catheter-associated infections have been utilized to examine the capacity of DGC inhibitors to reduce the bacterial load and biofilm persistence. For example, Sambanthamoorthy et al. (2014) demonstrated that DGC inhibitors effectively prevented biofilm formation on urinary catheters in a rat model and significantly reduced the bacterial burden in a murine lung infection model.

Pharmacokinetic profiling of DGC inhibitors is crucial for determining dosing regimens and optimizing therapeutic efficacy. Initial studies indicate that these compounds exhibit rapid systemic clearance but can be formulated to achieve sustained tissue levels, particularly in infection-prone sites such as the lungs and urinary tract (Sambanthamoorthy et al. 2014). Safety assessments further indicate that DGC inhibitors exhibit minimal cytotoxicity to mammalian cells at concentrations that are effective against biofilm formation, aligning with their targeted mechanism of action.

Importantly, because DGC inhibitors target virulence rather than viability, their impact on the host microbiome is expected to be less disruptive than that of conventional antibiotics. Early investigations suggest that these inhibitors do not significantly alter commensal bacterial populations, underscoring their potential for selective targeting of pathogenic biofilms without broad-spectrum microbiome disruption.

The development of resistance remains a critical consideration in the evaluation of DGC inhibitors. Serial passage experiments involving repeated exposure of bacterial populations to subinhibitory concentrations of DGC inhibitors have shown a lower propensity for resistance development than traditional antibiotics do, supporting the notion that antivirulence strategies may provide a more sustainable approach to infection management.

The future of DGC inhibitor development is moving toward more personalized therapeutic strategies, leveraging patient-derived tissues and organoids to assess drug efficacy in individualized infection models. While large-scale studies remain limited, integrating organ-on-chip technologies and ex vivo tissue platforms offers promising avenues for tailored infection control and identifying patient-specific therapeutic windows.

#### Potential clinical applications of diguanylate cyclase inhibitors

On the basis of preclinical data, some clinical uses for DGC inhibitors look especially promising. These proposed uses range from different types of infections to treatment modes and represent the widespread utility of biofilm formation in bacterial pathogenicity.

Chronic respiratory infections in cystic fibrosis (CF) patients are a primary target for DGC inhibitors. *P. aeruginosa* biofilms in CF airways are highly recalcitrant to treatment with standard antibiotics, resulting in relentless lung damage and worsening respiratory function. Studies have shown that nebulized inhalation of a DGC inhibitor enhances the effectiveness of tobramycin in a murine model of *P. aeruginosa* lung infection, potentially providing a rationale for combination therapy in CF patients (De Craemer 2023; Hamed & Debonnett 2017).

Catheter-associated urinary tract infections (CAUTIs), which are predominantly caused by biofilm-forming *Pseudomonas aeruginosa*, represent a significant clinical challenge, particularly in hospitals. These infections are highly resistant to conventional antibiotics, necessitating novel therapeutic approaches. Targeting the cyclic di-GMP (c-di-GMP) signaling pathway, which regulates biofilm formation, offers a promising strategy for disrupting established biofilms and promoting bacterial dispersion. Recent studies have shown that disperazol, a hydrochloride salt formulation of the c-di-GMP-degrading activator H6-335-P1, effectively reduces biofilm biomass and enhances antibiotic penetration. The combined administration of Disperazol and ciprofloxacin significantly improved treatment outcomes in a murine CAUTI model (Hultqvist et al. 2024).

Recent studies utilizing in silico pharmacophore screening have identified four diguanylate cyclase (DGC) inhibitors, LP 3134, LP 3145, LP 4010, and LP 1062, that effectively disrupt biofilm development on urinary catheters. These compounds reduce c-di-GMP levels, inducing biofilm dispersion and preventing biofilm reformation. Notably, these two inhibitors exhibited minimal toxicity to mammalian cells, highlighting their potential as adjunctive therapies for preventing and treating CAUTIs (Sambanthamoorthy et al. 2014).

In addition to these targeted uses, DGC inhibitors may also be used as adjuvants to standard antibiotic treatment for other biofilm-related infections. Through increased antibiotic penetration, bacterial susceptibility, and disruption of mature biofilms, these agents may enhance treatment efficacy for infections that are not responsive to antibiotics alone. Such an adjuvant strategy might be especially useful in treating infections caused by multidrug-resistant pathogens, where maintaining the effectiveness of available antibiotics is important.

#### Challenges and considerations for clinical translation

Although promising preclinical findings exist, several issues must be overcome in translating DGC inhibitors to clinical use. These issues cut across different areas, ranging from scientific to regulatory and commercial drug development.

Target selection is a significant factor due to the multiplicity of DGCs across most bacterial species. Most pathogenic bacteria harbor multiple DGCs whose functions may overlap or be redundant. For example, *P. aeruginosa* contains approximately 40 GGDEF domain-containing proteins (Eilers et al. 2024; Pestrak & Wozniak 2020). Such redundancy can make using an inhibitor against a specific DGC less effective because other enzymes may serve as backups for the inhibited enzyme. Several small-molecule DGC inhibitors (e.g., sulfasalazine, eprosartan, and sulfathiazole) that inhibit DGC activity and reduce biofilm formation in *Pseudomonas aeruginosa* and *Escherichia coli* have been identified (Römling et al. 2013; Sambanthamoorthy et al. 2014). However, these inhibitors often show variable potency across different DGC isoforms and bacterial species, and true broad-spectrum DGC inhibitors have not yet been identified in the clinic.

There is a need for cooperation between developers, regulatory authorities, and clinical specialists to define proper development paths. The commercial attractiveness of DGC inhibitors is subject to the same challenges as all antimicrobial development, such as small market size, price limitations, and uncertain investment returns. The possibility of using DGC inhibitors as adjuvants instead of single treatments makes their commercial application even more problematic. However, their potential to treat infections not well treated by standard antibiotics can open niche markets with less competition and a greater willingness to pay. Moreover, new business models, such as subscription-based payment schemes being tested in some nations, can alleviate the commercial difficulties of antimicrobial drug development.

Patient selection and consideration in diagnosis will be important in clinical trials and subsequent clinical application of DGC inhibitors. Owing to their mechanism of action, they may be maximally effective for treating infections that involve biofilms. Nevertheless, regular diagnostic techniques available to detect biofilms in clinical samples are suboptimal. Recent studies have developed methods for rapidly detecting cyclic di-GMP in clinical samples that could be used as a companion diagnostic for selecting patients most likely to benefit from DGC inhibitor therapy (Capatina et al. 2024; Nair et al. 2017). Integration into clinical practice and trials would enable a precision medicine approach to treating biofilm-associated infection.

#### Future perspectives and emerging trends

The field of DGC inhibition for combating bacterial biofilms continues to evolve rapidly, with several emerging trends and future directions worthy of consideration. The development of inhibitors that target diguanylate cyclases (DGCs) represents a promising frontier for combating *Pseudomonas aeruginosa* biofilm-associated infections. As key enzymes in the synthesis of cyclic di-GMP (c-di-GMP), DGCs regulate critical transitions between planktonic and biofilm lifestyles, making them attractive antivirulence targets (Valentini & Filloux 2016). Inhibiting DGC activity can impair biofilm maturation, surface adherence, and virulence factor production without exerting the intense selective pressure that typically accompanies bactericidal antibiotics(Kim et al. 2021). Furthermore, given the variability in DGC and phosphodiesterase (PDE) expression across strains, tailored inhibitor cocktails may offer more robust control over c-di-GMP levels in different clinical isolates (Valentini & Filloux 2016, 2019).

Recent studies have identified small-molecule inhibitors that reduce intracellular c-di-GMP levels, attenuate biofilm formation and promote biofilm dispersal. Compounds such as ebselen oxide and its analogs have shown promising results in inhibiting *P. aeruginosa* biofilm formation and reducing alginate production (Kim et al. 2021), a phenotype often associated with c-di-GMP-mediated regulation, although the precise mechanistic link to c-di-GMP remains to be fully elucidated.

The use of such antivirulence compounds may be further enhanced through combination therapies. For example, integrating DGC inhibitors with quorum sensing (QS) blockers or efflux pump inhibitors could theoretically amplify virulence suppression and delay resistance development, an approach conceptually aligned with the antivirulence strategies discussed by García-Contreras et al. (2016) (Garcia-Contreras et al. 2016). Additionally, DGC inhibition may resensitize bacteria to antibiotics, potentially restoring the efficacy of otherwise ineffective agents in biofilm-dense infections.

The targeting of specific DGC isoforms involved in motility regulation, such as WspR, *SadC*, and *YfiN*, could allow the selective disruption of pathogenic traits while minimizing off-target effects on beneficial microbiota (Katharios-Lanwermeyer et al. 2022; Malone et al. 2007; Merritt et al. 2007; Valentini & Filloux 2016). Moreover, DGCs have been implicated in regulating interbacterial competition and host colonization strategies, expanding their role as emerging therapeutic targets in broader microbiome-related and agricultural contexts (Lai et al. 2025).

As nanoparticle-based drug delivery systems advance, the encapsulation of DGC inhibitors in inhalable formulations may provide sustained release and targeted delivery to infected lung tissues in cystic fibrosis and ventilator-associated pneumonia (Hamed & Debonnett 2017; Sharma et al. 2021). These formulations can improve drug penetration into biofilms, enhance local retention, and reduce systemic toxicity.

Despite this potential, significant challenges remain. The redundancy and complexity of c-di-GMP signaling, involving multiple DGCs and effector proteins, necessitate a systems-level understanding of signaling networks and feedback loops (Römling et al. 2013). However, the development and clinical validation of such biomarkers remain in the early stages.

From a regulatory standpoint, evaluating antivirulence agents that do not reduce the bacterial burden in conventional models poses hurdles in demonstrating clinical efficacy. Nonetheless, their ability to disarm pathogens without applying intense selective pressure represents a paradigm shift in antimicrobial development.

Future research should prioritize the identification of potent, selective, and pharmacokinetically favorable DGC inhibitors. Structure-guided drug design, in combination with high-throughput screening of natural and synthetic libraries, will accelerate the discovery of lead candidates. Moreover, understanding the interplay between c-di-GMP signaling and other stress response pathways, such as quorum sensing, oxidative stress, and nutrient limitation, may offer new synergies for combination therapy. Ultimately, integrating DGC inhibitors into a multifaceted treatment strategy offers a promising avenue to outpace the evolution of resistance and effectively manage chronic *P. aeruginosa* infections.

## Conclusions and Outlook

Progress in developing DGC inhibitors as antibiofilm compounds has been significant over the last decade, from proof-of-concept investigations to preclinical testing in appropriate infection models. These developments have benefited from enhanced knowledge of cyclic di-GMP signaling pathways, novel high-throughput screening strategies, and advanced structural biology methodologies. Preclinical evidence for the promise of these compounds is strong, especially for indications where traditional antibiotics are hampered by biofilm formation.

Several important benefits of DGC inhibitors have been identified in preclinical studies, such as their effectiveness against mature biofilms, synergy with traditional antibiotics, activity against antibiotic-resistant bacteria, and potentially lower likelihood of resistance formation. These benefits place DGC inhibitors as strong contenders for tackling the increasing problem of biofilm-related infections, which substantially burden morbidity, mortality, and healthcare expenditures.

However, substantial hurdles still exist along the road to clinical translation, such as optimizing pharmacokinetic profiles, creating suitable formulations for specific uses, overcoming regulatory approaches tailored primarily to traditional antimicrobial drugs and achieving commercial feasibility in a challenging market environment. These hurdles must be overcome through cooperative endeavors by academic researchers, pharmaceutical firms, regulatory agencies, and clinical practitioners.

The area of DGC inhibition for treating bacterial biofilms is on the verge of ongoing growth and development. New trends, such as combination strategies, sophisticated delivery systems, individualized treatment approaches, and new targeting mechanisms, suggest fertile ground for ongoing research and development. With ongoing investment and attention, DGC inhibitors could realize their potential as valuable additions to the antimicrobial tool chest, responding to an unmet need in managing infectious diseases.

## Data Availability

No datasets were generated or analysed during the current study.
